# Basil seed mucilage as a bioadhesive polymer: Development of naproxen sodium microspheres and suppositories with *in-vitro* and *ex-vivo* studies

**DOI:** 10.5599/admet.2372

**Published:** 2024-10-03

**Authors:** Devika Tripathi, Krislay Rathour, Prashant Pandey, Ritesh Kumar Tiwari, Awani Kumar Rai

**Affiliations:** 1 PSIT-Pranveer Singh Institute of Technology (Pharmacy), Kanpur-209305, U.P., India; 2 Department of Pharmaceutical Sciences, Babasaheb Bhimrao Ambedkar University Lucknow- 226025, U.P., India; 3 Faculty of Pharmacy and Pharmaceutical Sciences, University of Alberta, Edmonton, Alberta T6G 2E1, Canada; 4 Shri Ram Murti Smarak College of Engineering and Technology (Pharmacy), Bareilly-243202, U.P., India

**Keywords:** *Ocimum basilicum*, mucilage, bioadhesive, drug solubility, colorectal administration, drug delivery

## Abstract

**Background and purpose:**

The study explores basil seed mucilage as a bioadhesive carrier for naproxen sodium, demonstrating its ability to enhance solubility when administered rectally. The mucilage, derived from *Ocimum basilicum* seeds, showed bioadhesive properties and thermal stability, as confirmed by FTIR spectroscopy and X-ray diffraction analysis.

**Experimental approach:**

Microspheres were prepared using a double emulsion solvent evaporation technique, varying polymer ratios to optimize drug delivery.

**Key results:**

Particle size analysis revealed a range of 456±0.51 to 712±0.21 μm, with larger microspheres formed at higher mucilage concentrations due to increased viscosity. Encapsulation efficiency ranged from 45.01±0.25 % to 79.4±0.93 %, improving with higher basil/alginate ratios. The superior batch, OBM5, showed excellent mucoadhesive qualities in *ex-vivo* assays, attributed to the increased polymer content, facilitating interaction with rectal mucosa. SEM analysis of OBM5 indicated a spherical, monolithic structure conducive to free flow. Drug release was efficient, with OBM5 achieving 88.7±1.3 % after 7 hours, indicating a controlled release profile.

**Conclusion:**

Incorporated into polyethylene glycol (PEG) 4000 suppositories, supposetories were completely disintegrated in buffer solution within 25 minutes. The bioadhesive force of basil seed mucilage on rectal mucosa was significantly enhanced, reaching 6.44±0.58 g, correlating with mucilage concentration. These findings underscore the efficacy of basil seed mucilage as a bioadhesive biopolymer for rectal drug delivery systems.

## Introduction

The rectum, an integral segment of the large intestine, serves as a conduit and transient reservoir during defecation. Its primary function involves minimal absorption of water and electrolytes. Accumulation of faecal matter within the rectal lumen persists until rectal distension elicits the defecation reflex. Notably, the rectum diverges from other gastrointestinal components by maintaining a stable milieu characterized by a neutral pH and constrained buffering capacity. Unlike the small intestine, which boasts absorptive villi and microvilli, the rectum exhibits limited absorptive capacity. Consequently, drug absorption predominantly occurs in the expansive surface area of the small intestine. Challenges inherent in oral drug administration encompass taste perception, gastric irritation, and the initial hepatic metabolism (first-pass effect). Pertinent gastrointestinal factors, including luminal pH, transit time, mucus composition, and metabolic enzymes, substantially influence drug stability and permeability. Furthermore, interindividual variability underscores the complex interplay of these factors [1]. In rectal pharmacotherapy, drug molecules are uptake via transcellular or paracellular pathways. This route offers a strategic advantage by partially circumventing the hepatic first-pass effect. The superior rectal vein channels blood from the proximal rectum to the portal circulation, leading to hepatic bioprocessing. In contrast, the middle and inferior rectal veins facilitate systemic absorption by directly merging with the inferior vena cava, thus bypassing hepatic filtration. Nonetheless, the extensive vascular anastomoses present can modulate this absorption route, influencing the pharmacokinetics of rectally administered drugs [2].

In drug delivery, natural products like mucilages and gum exudates are gaining prominence as pharmaceutical excipients. These plant-derived substances offer easy availability, compatibility, biodegradability, safety, and potential functional modifications. Among them, basil seeds (from *Ocimum basilicum*) stand out. These annual herb seeds thrive in the regions of Pakistan, India, and Iran. Researchers have explored basil seed powder as an excipient, evaluating its physiochemical properties, water-holding capacity, and phytochemical characteristics. The mucilage adhered to basil seeds exhibits remarkable hydration capacity. These hydrocolloids are promising carriers for enhancing the solubility and dissolution of poorly soluble drugs, particularly those in the Biopharmaceutics Classification System (BCS) class II. However, the safety and toxicity aspects of these polymers require further study [3].

In pharmaceutical research and development, augmenting a drug’s solubility constitutes a pivotal challenge [4]. Historically, a multitude of strategies have been utilized to increase the solubility of pharmaceuticals such as naproxen. These strategies encompass techniques like micro emulsification [5], solid-state dispersion [6,7], salt crystallization [8], pH adjustment solubilization [9], crystalline structure modification, nano emulsification, molecular inclusion complexation, and hydrotropic solubilization. Each method aims to enhance the aqueous solubility, thereby improving the bioavailability and therapeutic efficacy of the drug [10].

Recently, researchers have explored targeted drug delivery using novel nano-particulate carriers [11,12]. These carriers offer advantages such as site-specificity, improved drug stability through encapsulation, enhanced efficacy, reduced dosing frequency, and minimal side effects [13-15]. Micro/nanocarriers have shown promise for colon-specific drug delivery. Microspheres, which are tiny spherical structures ranging in size from 1 to 1000 μm, consist of both biodegradable and non-biodegradable materials. These microspheres exhibit prolonged residence times, allowing them to directly interact with the underlying absorption surface and enhance the effectiveness of therapeutic drugs [16,17]. Mucoadhesion facilitates precise drug delivery, ensuring optimal drug concentrations at sites beyond the intended target. Additionally, it safeguards labile compounds before, during, and after administration, prior to their arrival at the site of action. However, manufacturing microspheres presents challenges related to polymer properties, reproducibility, consistency, and filtration [18].

The pharmaceutical industry is persistently challenged by the issue of limited drug absorption, primarily due to poor solubilization and breakdown within the gastrointestinal tract. This limitation often necessitates higher doses to achieve the desired therapeutic effect, inadvertently increasing the risk of side effects, particularly in the gastrointestinal system where non-steroidal anti-inflammatory drugs (NSAIDs) are most active. Addressing the issue of poor drug solubility has thus become a priority in pharmaceutical research, leading to the exploration of innovative and enduring solutions [19]. One such approach that has garnered significant interest involves the use of natural polymers and excipients to enhance the solubility of poorly water-soluble medications. Mucoadhesive dosage forms have emerged as a crucial strategy in this context [20]. These forms facilitate improved drug absorption by prolonging the drug residence time at the absorption site through their attachment to the mucous membrane. The creation of bonds with the mucosal surface is aided by suitable carriers, one of which is a polymer derived from basil *Ocimum basilicum* seeds [21-23].

Naproxen, a non-steroidal anti-inflammatory drug synthesized from arylalkanoic acids, alleviates inflammation in joints afflicted by rheumatoid arthritis, osteoarthritis, and gout [24]. Its mechanism of action involves suppressing certain endogenous substances that promote inflammation. Naproxen’s solubility is exceedingly low, with a solubility measure of less than 0.2 μmol mL^−1^ in water, classifying it as practically insoluble. As a result, it is categorized under Class II of the Biopharmaceutics Classification System, which denotes drugs with low solubility in water yet high membrane permeability [25,26].

This study explores the application of basil seed mucilage as a mucoadhesive polymer in developing immediate-release Naproxen sodium microsphere suppositories. The encapsulation of the drug within microspheres precedes its integration into a suppository base. Recognized for its ability to enhance solubility, the mucilage underwent extraction and purification processes to ensure its compatibility with drug delivery systems. The characterization of the mucilage involved assessing its solubility, physical appearance, melting point, ash content, pH level, and swelling capacity. The formulation of Naproxen sodium microspheres utilized the mucilage, which was then subjected to optimization and evaluation through differential scanning calorimetry *(*DSC), FTIR and powder X-ray diffraction (pXRD). Additional analyses included measuring particle size and zeta potential, determining encapsulation efficiency, quantifying drug content, and calculating production yield. Subsequently, the microspheres were loaded with PEG 4000 to formulate the rectal suppositories. The suppositories' composition and stability were examined via *in-vitro* release and *ex-vivo* bioadhesion studies, followed by stability evaluations. The current research signifies the application of *Ocimum basilicum* mucilage in the rectal administration of Naproxen sodium, highlighting the mucilage’s potential as a mucoadhesive polymer in rectal drug delivery systems. The mucilage’s property of enhancing solubility may contribute to more favorable therapeutic effects and better adherence to treatment regimens.

This research investigates basil seed mucilage as a mucoadhesive polymer in the formulation of immediate release of Naproxen sodium microsphere suppositories. The study deviates from traditional rectal drug delivery methods by encapsulating the drug in microspheres prior to dispersion in a suppository base. The mucilage, known for its solubility-enhancing properties, was extracted and purified for suitability in drug delivery. Characterization parameters included solubility, appearance, melting point, ash value, pH, and swelling index. Naproxen sodium-loaded microspheres were formulated using mucilage, optimized, and evaluated using DSC, FTIR and pXRD. Determination of particle size and zeta potential, encapsulation efficiency, drug content, and production yield were also conducted. The microspheres were then incorporated into PEG 4000 to create rectal suppositories. The composition and stability of the suppositories were assessed through in-vitro release studies and ex-vivo mucoadhesion. Stability assessments were also conducted. This study represents the first use of *Ocimum basilicum* mucilage for Naproxen sodium rectal administration, highlighting the potential of the mucilage as a mucoadhesive polymer for rectal drug delivery. Its solubility-enhancing properties could lead to improved therapeutic outcomes and patient compliance.

Notably, this study represents the first utilization of *Ocimum basilicum* mucilage for Naproxen sodium rectal administration. Our investigation underscores the potential of basil mucilage as a versatile bioadhesive polymer for rectal drug delivery. Harnessing its solubility-enhancing properties paves the way for improved therapeutic outcomes and enhanced patient compliance.

## Experimental

### Materials

Naproxen sodium was procured from Yarrow Chem Products, Maharashtra, India. Seeds of *Ocimum basilicum L.* (Basil) were obtained from an established local market source and subsequently authenticated by a qualified botanist. Poly(ethylene glycol) (PEG) 4000 with 99 % purity was purchased from CDH Fine chemicals (New Delhi, India) and methanol with 99 % purity was acquired from Sigma Aldrich, India. All additional reagents and solvents employed were of analytical grade, ensuring the precision and reliability of experimental results.

### Extraction and solubility determination of Basil mucilage from seeds

The Ocimum basilicum seeds can produce mucilage when placed in water. This mucilage, which clings closely to the seed’s core, is an important source of both polysaccharides and soluble fiber. The extraction technique was adapted from the one described by Pradum Pundlikrao Ige et al. [27]. After washing 50 g of basil seeds to remove any dust and debris, a ratio of water to seeds of approximately 1:50 was used. The seeds were then left to soak and expand in distilled water heated to 65.1 °C for 2 hours. Mucilage removal was achieved by subjecting the seeds to mechanical agitation at 500 rpm for four hours. After swelling, the seeds were homogenized at a speed of 6000 rpm to facilitate the detachment of the mucilage. The isolated mucilage was then subjected to a purification process involving filtration through muslin cloth to eliminate minuscule seed particulates, followed by a centrifugation step at five minutes to remove any residual matter. The final stage entailed drying the mucilage under reduced pressure at temperatures ranging from 32 °C to 45 °C, after which it was pulverized into a fine powder and passed through an 80-mesh screen.

Further solubility studies of the mucilage were conducted in both aqueous and non-aqueous solvents by agitating dried mucilage in various solvents to assess its solubility properties. Additionally, the pH of the mucilage was determined by preparing a 1 % solution and measuring the pH using a digital glass electrode pH meter after full hydration.

#### Water-soluble ash value

The designated amount of ash was heated with 25 ml of water. Subsequently, the insoluble residue was separated using filtration, gathered on ash-free filter paper, and then rinsed with hot water. It was then incinerated in a pre-weighed crucible at a temperature not surpassing 45 °C for 4 hours. After cooling in a desiccator and weighing, the mass of the insoluble residue was deducted from the initial total ash weight. The resulting weight difference indicated the mass of the water-soluble ash component.

#### Acid insoluble ash

The quantification of acid-insoluble ash was conducted by subjecting the predetermined quantity of ash to boiling in 25 ml of 2 M hydrochloric acid for 5 minutes. After boiling, the non-soluble fraction was separated via filtration using an ash-free filter paper. This fraction was then thoroughly washed with heated water, followed by incineration within a tared crucible. The incineration process was executed at a controlled temperature not exceeding 45 °C for 4 hours. After the incineration, the residue was allowed to cool within a desiccator and weighed to ascertain its mass.

### Characterization of basil seeds mucilage

#### Total ash values

Ash values serve as a measure of a crude drug’s quality and purity, especially when it is in its powdered state. Ashing is crucial for eliminating all organic matter, which could disrupt the accuracy of analytical tests. When crude drugs are incinerated, they generally produce ash containing a mixture of sodium, potassium, calcium, and magnesium in carbonates, phosphates, and silicates. The percentage ash was calculated with reference to air-dried drugs using Equation (1):


(1)

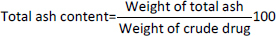



#### Phytochemical testing for carbohydrates and mucilage

To test the presence of carbohydrates, Molisch’s and Fehling’s tests were performed. For Molisch’s test, an aliquot of 2–3 mL from the aqueous extract was transferred into a test tube, followed by the addition of α-naphthol solution under constant agitation. After the initial steps, concentrated sulfuric acid drop by drop was added along the test tube’s side. This procedure led to a violet ring at liquid junction, indicating the presence of carbohydrates. Similarly, in Fehling’s test, 2 mL of mucilage was used. Equal parts of Fehling’s solutions A and B were added and heated for 10 minutes in a water bath. The appearance of a red precipitate was a positive confirmation of carbohydrates in the basil mucilage.

#### X-ray diffraction study

The X-ray diffraction (XRD) analysis of basil-derived mucilage was performed utilizing an Xpert3 Powder diffractometer system manufactured by Panalytical. The instrumental configuration incorporated 10 mm diverging and receiving slits for beam shaping. The operational parameters were set to a tube voltage of 40 kV and a tube current of 30 mA. The diffracted intensity data were collected over a 2-theta range spanning 10° to 90°. This methodological approach aligns with the procedures outlined in the research conducted by M.S. Hosseini *et al.* [28].

#### Thermogravimetric analysis

A thermogravimetric analysis (TGA) was performed on approximately 5 mg of basil seed mucilage using the SDTQ 600 TA apparatus to identify any potential polymorphic transitions in basil mucilage powder. The procedure entailed a scanning rate of 10° min^-1^ across a temperature spectrum of 50 to 300 °C within a controlled dry nitrogen atmosphere (N_2_).

#### Preparation and characterization of microspheres

Recent studies have highlighted sodium alginate as a potential carrier for drugs that are poorly soluble in water. It has been found particularly effective when used in the emulsification/gelation process to encapsulate drugs into microspheres smaller than 100 micrometers. These microspheres were developed through a specialized water-in-oil emulsification solvent evaporation method. The process began by creating a 1 % solution of basil seed mucilage and sodium alginate mixed with the drug. This mixture was then transferred into a volume of light liquid paraffin that included 0.5 %. Span-80 to act as an emulsifier. Using a magnetic stirrer, the mixture was then homogenized in an oil phase by agitating it at a steady 2000 rpm in a 500 ml beaker. The emulsion underwent crosslinking by being methodically added via a syringe with a 22-micrometer aperture. Following a designated period for crosslinking, the microspheres were purified with n-hexane and left to dry at ambient temperature. A total of five distinct drug-to-polymer formulations were produced, and the entire process was replicated three times to ensure reliability, as shown in Table 1.

**Table 1.** Drug-to-polymer selections for the microsphere’s preparation

Formulation		Content, mg		Crosslinking time, min	Stirring speed, rpm
Code	Naproxen sodium	Sodium alginate	Basil mucilage		
OBMF1	100	700	100	30	2000
OBMF2	100	500	200	30	2000
OBMF3	100	300	300	30	2000
OBMF4	100	200	500	30	2000
OBMF5	100	100	700	30	2000

#### FTIR study of microspheres

The FTIR spectrometer (PerkinElmer, Inc. US) was used to analyze the mucilage from O. basilicum, identifying various functional groups within the polymer. The analysis followed Thessrimuang et al. [29,30] analytical approach and investigated drug-mucilage interactions. Samples were ground and blended with anhydrous potassium bromide (KBr) in a 1:100 ratio. A motorized pellet press compressed the mixture into discs, ensuring adequate transmittance for the infrared analysis.

#### Differential scanning calorimetry study

The thermal properties of optimized microspheres were analyzed using differential scanning calorimetry (DSC). The DSC thermograms were obtained at a heating rate of 10 °C/min from 40 to 300 °C in a nitrogen atmosphere, flowing at 30 mL min^-1^ to ensure an inert environment during the analysis.

#### Particle size analysis, encapsulation efficiency and drug content analysis

The particle size analysis of the prepared microspheres was conducted using a Zeta sizer nano ZS90 (Malvern, UK), where the samples were prepared by re-dispersing the microspheres in distilled water. Each measurement was repeated three times for accuracy. For encapsulation efficiency, 100 mg of finely ground microspheres from each formulation were added to 250 mL of phosphate buffer (pH 6.8) and maintained at 37±0.5 °C for 24 hours with intermittent shaking. After incubation, the mixture was filtered through a 0.45 μm filter paper, and the absorbance of the filtrate was measured at 274 nm using a UV spectrophotometer (Shimadzu, Japan). The entrapment efficiency was calculated based on the ratio of the drug entrapped to the total drug content (Equation 2). Drug content was determined by mixing 100 mg of microspheres with 10 mL of purified water, followed by the addition of methanol and sonication for 3 minutes. The mixture was then filtered, diluted, and analyzed spectrophotometrically at 274 nm. The production yield of the microspheres was calculated by comparing the total mass of the microspheres with the initial mass of raw material, with the results expressed as a percentage representing the final yield on a dry weight basis (Equation 3).


(2)

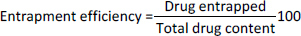




(3)

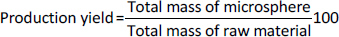



#### Determination of swelling index

A standardized protocol was implemented to assess the swelling index, which quantified the volumetric expansion characteristics of microspheres within a phosphate buffer at pH 6.8. Initially, a predetermined mass of 50 mg of microspheres was subjected to a swelling process for 24 hours at an ambient temperature of 25 °C within a pH 6.8 phosphate buffer. Following the completion of the swelling interval, any residual buffer was meticulously decanted, and the microspheres were subjected to a blotting procedure to eliminate excess moisture. The microspheres were then re-weighed to ascertain the net increase in mass of the microspheres. The swelling index was subsequently calculated utilizing the formula.


(4)

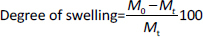



where *M*_t_ = microspheres initial weight and *M*_0_ = weight of microspheres at equilibrium swelling in the media.

#### Scanning electron microscopy study

The structural characteristics of microspheres loaded with naproxen sodium were investigated using a scanning electron microscope (Hitachi High Technology in Pleasanton, California). For this procedure, diluted microsphere samples were arranged on stubs and permitted to dry naturally. Afterward, they were coated with a gold layer to enhance conductivity and then scrutinized under the SEM for detailed observation.

#### *In-vitro* drug release

The release of naproxen sodium from various microsphere formulations was determined using a USP-II rotating basket-type dissolution test apparatus (HICON, New Delhi, India). Precisely weighed quantities of microspheres containing 100 mg of the drug were taken and the dissolution profile was ascertained in 900 ml of phosphate buffer (pH 6.8) at an agitation speed of 50 rpm and a controlled temperature of 37±0.5 °C. Periodic sampling involved the withdrawal of 5 ml aliquots at predetermined intervals, which were immediately replaced with an equivalent volume of pre-equilibrated dissolution medium to maintain sink conditions. The retrieved samples underwent filtration using 0.45 μm pore-size membrane filters. Quantitative analysis of naproxen sodium was conducted by measuring the ultraviolet absorbance at 274 nm, employing a calibrated UV-visible spectrophotometer. The resulting data were processed to calculate the cumulative percentage of drugs released, facilitating the assessment of the microsphere release characteristics.

#### *Ex-vivo* mucoadhesion study

The mucoadhesive characteristics of the microspheres were quantitatively evaluated employing a modified version of the protocol delineated by Ahmad *et al*. [31] and Nayak *et al.* [32,33]. The assay was conducted on a 5 cm segment of goat intestinal tissue, procured post-mortem from a local butcher within one hour of the animal’s demise. The tissue was meticulously cleansed with a physiologically isotonic saline solution. A 100 g of microspheres was uniformly distributed over the mucosal epithelium, affixed to a 20×10 cm transparent vitreous substrate. The assembly was positioned at an angle of 45 °C to the horizontal axis. A phosphate-buffered saline solution, adjusted to pH 6.8 and maintained at 37 ± 0.5 °C, flowed over the mucosal layer at a rate of 5 ml/minute. The time required for detaching all the microspheres from mucosal surface of goat intestine was recorded by visual inspection and mucoadhesion was calculated using equation (5):


(5)

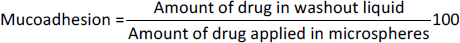



#### Statistical analysis

The experimental results were analysed using one-way ANOVA to assess a statistically significant difference between formulation batches defined as *p* < 0.05.

### Preparation and physicochemical characterization of microsphere-based suppositories

The fabrication of microsphere-embedded suppositories was executed employing the fusion method. This methodology necessitated the precise quantification of the polyethylene glycol (PEG) 4000 base, subsequently liquefied within a beaker at a controlled temperature of 40 °C via a water bath. Post-liquefaction, a predetermined quantity of the optimized OBM5 microspheres was amalgamated into the molten base under continuous agitation. Prior to the introduction of the molten admixture, the suppository molds were lubricated using glycerin to facilitate the subsequent release. The homogenized mixture was then carefully dispensed into the molds. After filling, the molds were subjected to a freezing environment at -4 °C to induce solidification. Upon achieving a solid state, the suppositories were gently extricated and underwent a comprehensive characterization to evaluate various performance metrics [34,35].

#### Physical testing

Randomly selected suppository samples underwent dimensional analysis using a digital Vernier caliper. The length and diameter of each sample were meticulously recorded. A longitudinal section was cut from each suppository to facilitate a visual examination to evaluate the homogeneity of the internal matrix in terms of texture and color.

Considering the critical impact of drug release kinetics on liquefaction, the melting characteristics of the suppositories were rigorously assessed. Each suppository was placed in a container with phosphate buffer (pH 6.8), maintained at a constant temperature of 37±0.5 °C. The time required for complete melting or dispersion of the suppository within the medium, referred to as the liquefaction time, was observed.

#### Determination of bioadhesive force

The epithelial lining of the rectum is often compared to the lining found in the upper part of the digestive tract, and drugs typically cross the rectal mucosa through transcellular permeation. However, when administering drugs in the rectal mucosa, it’s important to measure the bioadhesive force of the formulation. Therefore, to administer suppositories embedded with microspheres, we determined the bioadhesive force [36].

In this study, a well-established methodology was employed for the analysis. Rat rectums were prepared by subjecting them to a day of fasting with access only to sterilized double-distilled water. The rectums, once fresh, were cleansed with SCF and refrigerated at 4 °C. Suppositories containing microspheres were sectioned into 0.1 g pieces with a 0.5 cm diameter and affixed to probes. Rectal samples, approximately 2 cm^2^ in size, were positioned on the load platform. The colorectal mucosa received an addition of 100 ml of SCF. A Texture Analyzer was utilized to assess the bioadhesive force at 37 °C, measuring both the minimum bioadhesive force, *F*_min_, and the detachment stress (grams), which was the least weight required to separate the colorectal mucosa from the suppository. This study was performed in collaboration with Shri Ram Murti Smarak College of Engineering and Technology (Pharmacy), Bareilly and received approval from the Animal Ethical Committee /CPCSEA, under the approval number 715/02/c/CPCSEA.

#### *In-vitro* drug release

The in vitro drug release from suppositories was assessed using a USP type II paddle dissolution apparatus. The dissolution media were maintained at a temperature of 37 ± 0.5 °C and a speed of 50 rpm, utilizing 100 mL of a phosphate buffer solution with a pH of 6.8. Samples of 5 mL were collected at appropriate time intervals (from 0 to 25 min), and the drug concentration was measured spectrophotometrically at 274 nm. These measurements were conducted in triplicate.

## Results

### Phytochemical screening tests for extracted mucilage

Upon drying and coarse grinding, the seeds of *O. basilicum* produced a substantial yield, amounting to 30 wt.% of dry mucilage.

The aqueous seed mucilage dispersion exhibits a pH value 6.45, indicating a slightly acidic environment. This pH range aligns with the optimal conditions for biodhesion properties [37,38].

The solubility of basil mucilage was investigated in various solvents. Distilled water demonstrated solubility, while other solvents rendered it insoluble. In the subsequent phytochemical analysis, the presence of carbohydrates was confirmed using specific reagents, yielding positive results in the mucilage test. Similarly, the ash value analysis indicated the complete removal of extraneous or organic matter, affirming that the mucilage was non-toxic. Additionally, acid treatment effectively eliminated inorganic impurities such as phosphates, silicates, and carbonates. Furthermore, the low levels of total ash and acid-insoluble ash demonstrated minimal contamination during collection and handling. Table 2 given below, shows all physicochemical screening results.

**Table 2.** Results of screening tests of Basil seed mucilage

Tests	Observations
Solubility study	
Distilled water	Freely soluble
Ethanol	Practically insoluble
Acetone	Insoluble
Chloroform	Insoluble
Phytochemical screening test	
Carbohydrate test	
Molisch test	Confirmed the presence the carbohydrate due to presence of violet ring at the junction
Fehling’s test	Confirmed due to formation of red precipitate
Mucilage Test	
Water	Confirmed due to swelling of extracted basil powder
Using Ruthenium red	Confirmed the mucilage presence due to presence of red color
Ash values	
Content of total ash	7.4 %
Content of water-soluble ash	0.38 %
Content of acid-insoluble ash	1 %

#### Thermogravimetric study

**Figure 1.**
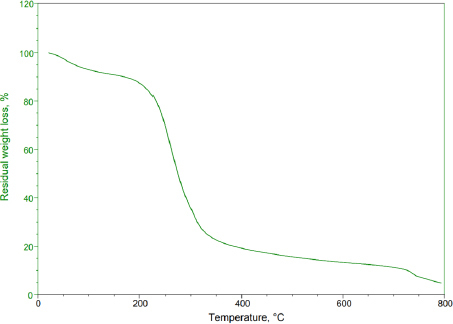
TGA curve of Basil seed mucilage

Thermogravimetric analysis is an essential method in polymer identification. This technique quantifies the mass variation of a substance in relation to temperature within a controlled environment. It yields valuable data on the thermal stability, oxidation tendencies, and compositional characteristics of materials. When applied to basil seed mucilage, TGA profiles were documented across a temperature spectrum from 50 to 800 °C at a consistent heating pace of 10 °C min^-1^ in a nitrogen environment. The basil seed mucilage demonstrated thermal decomposition within these thermal conditions. The initial weight loss occurred at 56 to 124 °C, corresponding to a 4 % reduction in weight. Subsequently, at 218 to 394 °C, approximately 49 % weight loss occurred due to further thermal degradation. The range from 428 to 479 °C witnessed continuous decomposition of basil seed mucilage. Figure 1 shows the TGA curve of thermal degradation of basil seed mucilage.

### Characterization of Naproxen sodium-loaded microspheres by FTIR

The FTIR spectrum was recorded for individual polymers and microspheres to determine the interaction between the drug and polymer.

**Figure 2.**
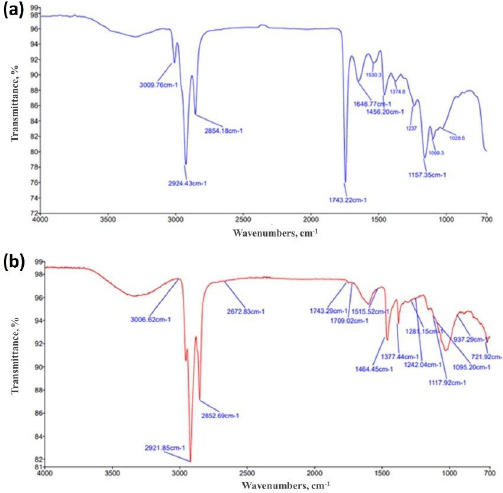
(a) FTIR of basil seed mucilage; (b) Basil seed mucilage loaded microsphere

The FTIR spectra of basil seed mucilage absorbance band at 3009 cm^-1^ corresponds to OH stretching, indicating the presence of hydroxyl groups, which are common in polysaccharides and important for water interactions and solubility. The bands at 2924 and 1646 cm^-1^ were attributed to CH_2_ stretching, which is part of the backbone structure of organic compounds and carbohydrates. The band at 1626 cm^-1^ corresponds to asymmetric stretching of carboxylate groups, often found in uronic acids, which are components of mucilage. The band at 1456 cm^-1^ indicates the presence of protein within the mucilage. The band at 1157 cm^-1^ corresponds to C–O–C stretching, supporting the presence of glycosidic linkages in the mucilage. The occurrence of the COO- group specifically signifies the presence of uronic acid in basil seeds, contributing to the mucilage's water-holding capacity and viscosity, whereas the band at 1026 cm^-1^ indicates the presence of arabinose in the mucilage. Similarly, various distinct bands were observed in the FT-IR spectra of basil seed mucilage-loaded microspheres. The bands at 3009 and 1242 cm^-1^ correspond to C_6_H_6_ group stretching and hydroxyl group O–H stretching, respectively. Bands at 1464, 1377, 1243 and 1281 cm^-1^ were associated with asymmetric stretching of alkanes. A broad band at 3009 cm^-1^ indicated aromatic ring vibrations, while another band at 2921 cm^-1^ was attributed to alkanes, indicating the compatibility of basil seed mucilage with naproxen sodium. Figures 2(a) and 2(b) show the obtained frequencies band in FTIR spectra of basil seed mucilage and mucilage-loaded microsphere.

#### Differential scanning calorimetry study

**Figure 3.**
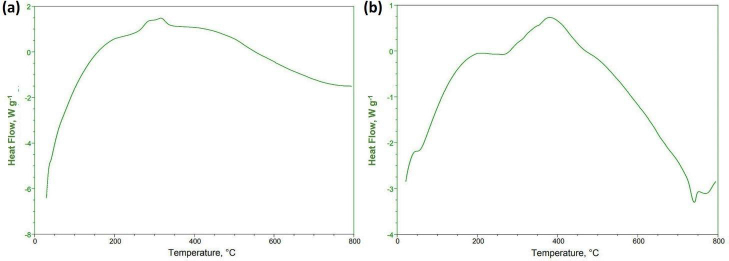
DSC curves (a) Basil seed mucilage (b) Optimized OBMF5

Basil seed mucilage showed intriguing behaviour during heating, with exothermic peaks indicating the formation of a polymer network shown in Figure 3(a). The glass transition temperature (*T*_g_) occurred at a lower onset temperature, causing the material to shift from a rigid, brittle state to a more flexible, rubbery state. Polysaccharides played a pivotal role in this transition, as they lost their crystalline structure, resulting in a disorderly arrangement. A DSC study of selected OBMF5 microspheres showed an endothermic peak at 75 °C, corresponding to its known melting point of 77.6 °C as shown in Figure 3(b). As the temperature increased, the enhanced formulation displayed a substantial endothermic peak, but this diminished, indicating a change in the material's thermodynamic properties. The formulation undergoes an exothermic reaction at 385 °C, releasing heat, and this continued until 432 °C when a broad peak emerged. Despite these temperature fluctuations, the sample demonstrated excellent stability, with a more significant endothermic peak, suggesting the formulation can withstand elevated temperatures without significant degradation. Therefore, it is revealed that there was no interaction between excipients and drugs [39-41].

#### Powder X-ray diffraction study

**Figure 4.**
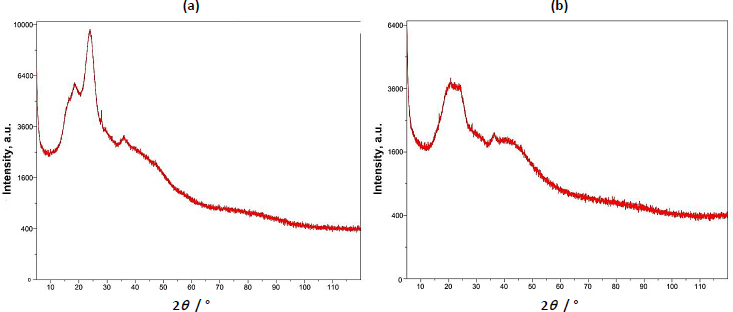
pXRD curves (a) Basil seed mucilage (b) OBMF5 microsphere

The pXRD study of basil seed mucilage revealed a broad peak around 22.90°, indicating an amorphous structure with low crystallinity. The absence of sharp peaks suggests an amorphous structure. Similarly, the presence of a broad peak in the 22-25° range in microsphers suggests the microsphere formulation did not affect the mucilage's amorphous nature, suggesting molecular dispersion of the drug in the polymer [42,43]. Figure 4 (a) shows a pXRD study of basil seed mucilage and (b) pXRD curve of OBMF5 microsphere.

#### Particle size, production yield, encapsulation efficiency and drug content determination

**Figure 5.**
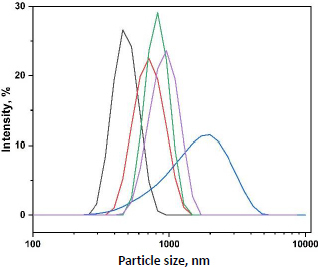
Particle size analysis of prepared microspheres

When basil mucilage was integrated into the alginate polymer solution, microsphere dimensions showed a noticeable enlargement. Concurrently, the viscosity of the polymer matrix solution increased, which, upon mixing with the crosslinking solution, produced larger droplets [44,45]. Consequently, the size of the microspheres was determined to be between 456 ± 0.51 and 712 ± 0.21 μm. Figure 5 shows the particle size distribution of prepared microspheres.

The production yield of microspheres (labeled OBMF1 to OBMF5) ranged from 32.16±0.23 to 84.27±0.13 % is presented in Table 3. As the drug-to-polymer ratio increased, the production yields gradually improved. Notably, the highest entrapment efficiency was achieved when mucilage was used in combination with alginate as a matrix network. This combination likely prevented drug leaching during the crosslinking process. Losses incurred during microsphere hardening, washing, and filtering may have contributed to overall drug loss [46,47].

**Table 3.** Combined results of actual drug content, production yield, encapsulation efficiency and mucoadhesion

Formulation code	Actual drug content, %	Production yield, %	Encapsulation efficiency, %	Mucoadhession, %
OBMF1	26.21 ± 0.16	32.16± 0.23	45.01 ± 0.25	48.28±0.32
OBMF2	38.45± 0.17	46.32 ± 0.21	52.43 ± 0.29	53.45±0.24
OBMF3	53.51 ± 0.23	52.48± 0.65	56.72 ± 0.26	66.23±0.51
OBMF4	65.23 ± 0.31	64.54 ± 0.42	65.45 ± 0.51	71.15±0.12
OBMF5	78.12 ± 0.43	84.27 ± 0.13	79.43 ± 0.93	81.79±0.22

The actual drug content in the microspheres exhibited a range from 26.21±0.16 to 78.12±0.43 % (Table 3). Notably, a negative correlation exists between drug loading and polymer concentration. As the polymer concentration increases, the drug loading capacity decreases. This phenomenon arises due to the limited space available for drug molecules within the polymer matrix. As the polymer chains occupy more volume, they leave less room for drug incorporation. Consequently, the overall drug content in the microspheres was affected.

#### *In-vitro* mucoadhesion study

The *in-vitro* mucoadhesion tests revealed that all the formulated microspheres had displayed adequate mucoadhesive characteristics, with values ranging from 48.28 ± 0.32 to 81.79 ± 0.2 % (Table 3). The increase in polymer concentration corresponded to a higher rate of mucoadhesion, which was attributable to the greater proportion of polymer available for interaction with the mucosal layer.

#### Swelling index

The swelling index results for the microspheres demonstrated that basil seed mucilage had a superior absorption capacity. The heightened absorption capacity stemmed from its outstanding ability to retain water within the polysaccharide matrix, notably within capillary structures. Moreover, water molecules formed hydrogen bonds and dipoles with the molecular components of the soluble fiber. However, an increase in the percentage of basil seed mucilage within the microspheres led to a reduction in the swelling percentage. This reduction was due to the formation of stronger bonds between sodium alginate and basil seed mucilage, which resulted in smaller cavities. The observed gradual growth trend indicated that microspheres with different weight percentages of basil seed mucilage showed a slower water absorption rate. Table 4 shows the mean swelling index of prepared microspheres.

**Table 4.** Swelling index of microspheres

Formulation	Initial weight of microspheres, mg	Final weight after swelling in media, mg	Degree of swelling index	Mean swelling index (±SD)
OBMF1	100	120	16.66±0.011	16.56±0.034
OBMF2	100	130	23.07±0.034	23.78±0.012
OBMF3	100	141	29.07±0.015	29.72±0.027
OBMF4	100	148	32.43±0.024	33.93±0.051
OBMF5	100	157	36.30±0.051	37.64±0.038

#### Scanning electron microscopy study

**Figure 6.**
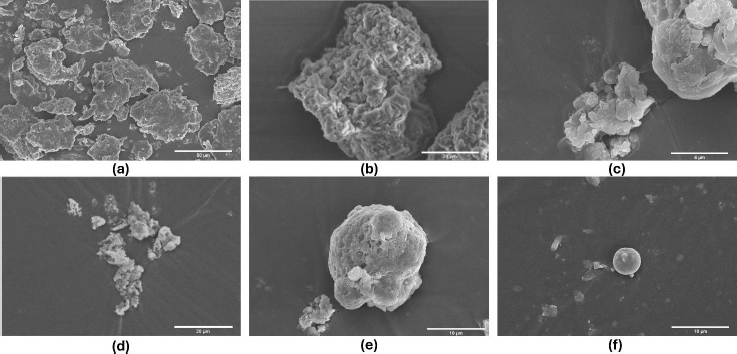
Surface morphology study of (a) basil seed mucilage; (b) OBMF1; (c) OBMF2; (d) OBMF3; (e) OBMF4; and (f) OBMF5

In the basil seed mucilage surface morphology study, samples were examined at 2000× magnification using an electron beam accelerated at 10.00 kV and captured using a high vacuum ETD (Hitachi High Technology in Pleasanton, California.). The particles displayed textured surfaces, suggesting either a porous structure or surface roughness. However, the spherical structure was more pronounced in OBM4 and OBM5. External morphology analysis revealed consistent production of well-defined spherical particles across all formulations (OBM1 to OBM5). These microspheres exhibited a sufficiently rough, wrinkled, and porous surface texture. Notably, the optimized formulation OBMF5 displayed a distinct spherical particle shape compared to OBMF1, OBMF2 and OBMF3 formulations. The OBM5 microspheres were characterized as discrete, spherical, and free flowing, belonging to the monolithic matrix type. This increase in mean particle size with higher polymer concentration likely resulted from increased viscosity, leading to larger emulsion droplets and, ultimately, larger microspheres [3,48]. The presence of basil mucilage in OBM5 significantly strengthened the microsphere structure, enhancing wall thickness and creating an ideal compound for drug release. Figure 6 shows the surface morphology of basil seed mucilage and prepared microsphere formulation.

#### *In-vitro* drug release

**Figure 7.**
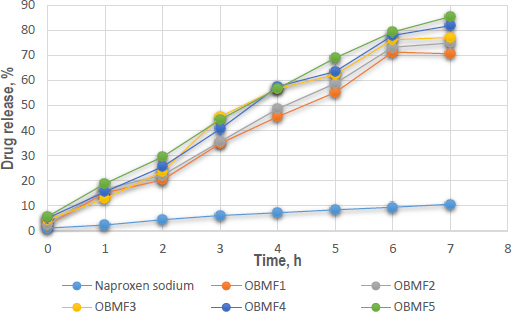
In-vitro drug release of naproxen sodium and prepared basil seed mucilage microspheres

In-vitro dissolution experiments were conducted on naproxen sodium encapsulated within basil mucilage microspheres using a phosphate buffer solution at a pH of 6.8, mimicking the small intestine's environment. The optimized formulation, OBMF5, showed the highest drug release, with 85.4 ± 0.21 % after 7 hours. The difference in release patterns was due to the increased amount of basil seed mucilage, which enhanced solubility and controlled the release of naproxen sodium. The hydrophilic nature of the mucilage formed a gel-like barrier around the drug, modulating its release rate. The initial release was higher for the microspheres, particularly OBMF4 and OBMF5, indicating faster burst release compared to pure naproxen sodium. The release rate continued to increase significantly for the microspheres, reaching 85.4 % at 7 hours [49-51]. The narrow error bars indicated good reproducibility. Figure 7 shows the *in-vitro* drug release of microspheres.

#### Statistical analysis

The corresponding p-value obtained for formulation batches was 0.0141. Since the p-value was less than 0.05, the results were statistically significant.

### Physicochemical evaluation of microsphere-loaded suppositories

The suppositories were prepared, cut longitudinally, and visually inspected for physical characteristics such as fissures, fat blooming, exudation, and active ingredient movement. Fortunately, no such issues were observed. The typical suppository dimensions are approximately 258±0.56 mm in length and 84±0.43 mm in breadth. The average weight of the 10 suppositories was 1.898 ± 0.98 g, meeting the Indian Pharmacopeia standards for weight variation. Regarding mechanical properties, the rectal suppositories exhibit a hardness ranging from 1.0 to 0.64 kg cm^-2^ and possess a smooth and fine texture. Their fragility or brittleness, which affects their ability to withstand handling, packing, and transportation risks, was assessed through crushing or breaking strength. In the study, the suppository exhibited a friability of less than 1%, below the permissible threshold [52]. The dissolution test, performed in a buffer solution with a pH value of 6.8 and at a controlled temperature of 37.5 °C, resulted in the complete melting of the suppository within an average time of 24.4±0.37 minutes. After the 25-minute interval, the suppository was observed to have completely dissolved in the buffer medium.

#### *In-vitro* drug release of suppositories

**Figure 8.**
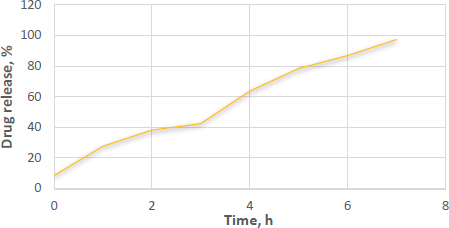
*In vitro* drug release of suppository

The suppositories melted in the dissolving media maintained at 37±0.5 °C, as observed during the dissolution study. The optimized formulations exhibited a drug release of 97.66 % after 7 h. Additionally, these suppositories completely melted within approximately 24.5±0.5 minutes. Interestingly, suppositories loaded with microspheres demonstrated a slight increase in drug release, as presented in Figure 8. This phenomenon could be attributed to the hydrophilic properties of PEG 4000.

#### Determination of bioadhesive force

A study was conducted to measure the bioadhesive force of an optimized suppository formulation on the rectal membrane. A higher percentage of basil mucilage led to an enhanced bioadhesive force, resulting in a value of 6.44 ± 0.58 g. These findings align with the Adsorption theory of Mucoadhesion, which suggests that two surfaces stick together through the action of surface forces related to their chemical makeup [53,54]. When polar entities are involved, they may reorient at the contact surface. If the adhesion is intense, chemisorption might take place. This theory proposes that the sticking of tissues is due to various secondary forces such as van der Waals forces, hydrogen bonds, and hydrophobic interactions [55,56].

## Discussion

Recently, mucilage derived from basil seeds has gained attention as an effective biopolymer for drug delivery systems. Derived from *Ocimum basilicum* seeds, this natural polymer offers several advantages, including biodegradability and cost-effectiveness. In our study, the authors investigated the potential of basil seed mucilage as a bioadhesive carrier for naproxen sodium. Combining basil seed mucilage with sodium alginate is aimed at enhancing drug solubility and improving rectal administration.

To assess the bioadhesive properties of the basil seed mucilage as polymer, basil mucilage from *Ocimum basilicum* seeds was obtained through a rigorous extraction process and evaluated by analytical techniques. An FTIR spectroscopy confirmed the presence of characteristic bands at 3009, 2924, 1626, 1456, 1374 and 1028 cm^-1^, corresponding to functional groups within the mucilage. Notably, a key component, uronic acid was detected, emphasizing the mucilage’s potential as a bioadhesive material. Similarly, in phytochemical tests using Molisch and Fehling’s reagent, the presence of carbohydrates in the mucilage was confirmed [57]. The pH of the aqueous seed dispersion was measured and found to be 6.45 (slightly acidic). X-ray diffraction analysis revealed an amorphous structure with very low overall crystallinity. Crystalline regions were observed at an angle (2 θ) of 23-25°, further validated by a powder X-ray diffraction study. The amorphous nature of basil seed mucilage was attributed to its glass transition temperature of 146 °C, which is considered relatively high. Polysaccharide chains within the mucilage fall out of their crystalline structure and become disordered during this transition. The thermal stability assessment revealed that basil mucilage remained thermally stable, with no evidence of polymorphic transitions. Furthermore, the quality and purity of basil mucilage were validated through measurements of total ash, water-soluble ash, and acid-insoluble ash. The respective values were 7.4, 0.38, and 1 %. These low ash values indicate minimal contamination during collection and handling. In the TGA, three distinct steps were observed for basil seed mucilage. Initial Weight Loss (35 to 100 °C). This step corresponds to moisture vaporization. In the study, the initial weight loss for basil seed mucilage occurred at 58 to 121 °C. Basil seed mucilage experienced thermal degradation in this temperature range of 220 to 398 °C. Moreover, the third step involved continuous decomposition, resulting in weight loss. The weight loss percentages were 3.97, 48.11 and 6.57 %.

Thus, basil seed mucilage was utilized as a bioadhesive agent in the formulation of microspheres for rectal drug delivery systems designed to facilitate prompt drug release. The double emulsion solvent evaporation technique was employed to fabricate five distinct microspheres, maintaining a constant drug quantity while varying the polymer ratios. The resultant microspheres underwent comprehensive characterization, including particle size analysis, DSC, XRD, SEM, along with assessments of production yield, drug content, encapsulation efficiency, ex-vivo mucoadhesion, swelling index, and in-vitro drug release performance.

Polymer concentration significantly influenced the size, swelling, and entrapment of naproxen sodium microspheres. The microspheres’ size increased with the incorporation of basil mucilage into the alginate polymer solution due to the increased viscosity of the combined polymer solution, which enlarged the droplet size [58-60]. The size range was found to be 456±0.51 to 712±0.21 μm. Higher concentrations of basil seed mucilage yielded more viscous polymer solutions, requiring greater energy to break into smaller droplets, resulting in larger microspheres with an encapsulation efficiency ranging from 45.01±0.25 to 79.43±0.93 %. The drug content rose with an increase in the basil/alginate ratio, indicating that basil mucilage improved drug entrapment, with values ranging from 26.21±0.16 to 78.12±0.43 % from OBM1 to OBM5. Additionally, the FTIR spectrum confirmed the compatibility of basil seed mucilage with blank microspheres, showing no interaction between the drug and mucilage, thus deeming the mucilage suitable for microsphere formation. Swelling, a critical parameter for bioadhesive microspheres, was assessed, revealing maximum swelling up to 7 hours in basic media. The increased solubility of the polymer at a slightly acidic pH (6.8) led to the relaxation of the crosslinked polymeric network in the microspheres, resulting in enhanced swelling.

Upon evaluation of parameters such as particle size, encapsulation efficiency, yield of production, content of the active pharmaceutical ingredient, and the index of swelling, the OBM5 batch was determined to be the superior formulation. Analytical techniques, specifically XRD and DSC, were employed to elucidate the impact of the microencapsulation technique on the crystalline structure of the drug. Notably, the XRD patterns of OBMF5 lacked the distinctive bands indicative of naproxen sodium’s crystallinity, implying a transition to an amorphous state within the microsphere matrix. This transformation was further supported by the absence of crystalline domains in the XRD profile, suggesting a homogenous drug distribution. Scanning electron microscopy (SEM) analysis disclosed particles with a textured topology, potentially reflective of porosity or surface irregularities. The grayscale variance in the SEM images indicated material composition or density variations. Moreover, the spherical morphology was accentuated in formulations OBM4 and OBM5. Morphological examination revealed the generation of spherical entities with a rough, wrinkled, and porous texture, a trend consistent across the OBM series. The optimized OBMF5 formulation displayed a pronounced sphericity relative to its non-optimized counterparts [61-63]. The microspheres of OBM5 were characterized as distinct, spherical, and monolithic, with a propensity for free flow. An escalation in polymer concentration correlated with an increase in particle size, likely due to augmented viscosity and subsequent enlargement of emulsion droplets, culminating in increased microsphere dimensions. The OBMF5 formulation showcased a drug release of 88.7±1.3 % post a 7 h duration, surpassing all other microsphere formulations. The initial release was higher for the microspheres, particularly OBMF4 and OBMF5, indicating faster burst release compared to pure Naproxen sodium. *Ex-vivo* mucoadhesion assays confirmed the superior mucoadhesive qualities of the microspheres, with OBMF5 exhibiting the most pronounced mucoadhesion, attributable to the augmented polymer content facilitating interaction with the rectal mucosa. Consequently, the OBMF5 microspheres were integrated into polyethylene glycol (PEG) supposetories for further physicochemical evaluation.

In a rigorous physical evaluation, the suppositories demonstrated an absence of active ingredient migration, as well as a lack of fissuring, fat blooming, and exudation phenomena. Dimensional analysis yielded lengths and breadths of 25.8±0.56 and 84±0.43 mm, respectively. The mean mass of the suppositories was determined to be 1.898±0.98 g, aligning with the Indian Pharmacopeia standards, exhibiting less than a 5 % deviation according to the weight variation test. Mechanical integrity assessments revealed strengths ranging from 1.0 to 0.64 kg cm^-2^, complemented by a uniformly smooth and refined texture. In-vitro dissolution testing showcased a complete disintegration in buffer solution within 25 minutes due to the enhanced dissolution rate and the hydrophilic properties of PEG 4000. Furthermore, the bioadhesive potential of basil seed mucilage on rectal mucosa was quantified, revealing that an increased mucilage concentration significantly augmented the bioadhesive force to 6.44±0.58 g.

## Conclusions

Bioadhesive systems offer distinct advantages by forming strong interactions with mucosal tissues, which leads to longer residence time and sustained drug release, reducing the need for frequent dosing. This is crucial for enhancing patient compliance. Furthermore, the targeted delivery capabilities of these systems can result in both localized and systemic therapeutic effects. The use of *Ocimum basilicum* seed mucilage is particularly promising for improving pharmaceutical formulations and increasing drug solubility, potentially leading to better patient outcomes and a wider range of treatment options.
